# Behavior Change Techniques in Digital Health Interventions for Promoting Adolescent Health Behaviors: Systematic Umbrella Review

**DOI:** 10.2196/84754

**Published:** 2026-05-06

**Authors:** Nikolaos Boumparis, Philippe de Riedmatten, Katrina Champion, Gloria Cea, Teresa de Pablo-Pardo, Kleio Koutra, Katie Rizvi, Hayley Pearce, Andreas Triantafyllidis, Ana Molina-Barceló, Michael Patrick Schaub, Severin Haug

**Affiliations:** 1 Swiss Research Institute for Public Health and Addiction, University of Zurich, Konradstrasse 32, Zurich, 8005, Switzerland, +41444481173; 2 The Matilda Centre for Research in Mental Health and Substance Use, The University of Sydney, Sydney, Australia; 3 School of Public Health, The University of Sydney, Sydney, Australia; 4 BRIDG OÜ, Tallinn, Estonia; 5 Cancer and Public Health Research Unit, Foundation for the Promotion of Health and Biomedical Research, Valencia, Spain; 6 Laboratory of Applied Social Research and Social Work, Department of Social Work, Hellenic Mediterranean University, Heraklion, Greece; 7 Youth Cancer Europe, Cluj-Napoca, Romania; 8 Department of Communication Sciences, Ghent University, Ghent, Belgium; 9 Centre for Research and Technology Hellas, Thessaloniki, Greece

**Keywords:** behavior change techniques, digital health, adolescent health, health promotion, systematic review

## Abstract

**Background:**

Digital health interventions (DHIs) using behavior change techniques (BCTs) show promise in addressing adolescent health behaviors, but evidence of their effectiveness across health behavior domains remains fragmented and poorly summarized.

**Objective:**

This systematic umbrella review synthesized evidence from existing systematic reviews on the effectiveness of BCTs within DHI targeting key adolescent health behavior domains: alcohol consumption, tobacco use, physical activity, dietary habits, and obesity management.

**Methods:**

We systematically searched PubMed, PsycInfo, Embase, and CINAHL in April 2024 for reviews of DHI for adolescents (10‐19 years old). We coded all identified BCTs using the Behavior Change Technique Taxonomy version 1 (BCTTv1). Data on BCT effectiveness, intervention characteristics, and review quality were extracted and narratively synthesized using AMSTAR-2 (A Measurement Tool to Assess Systematic Reviews 2).

**Results:**

A total of 20 reviews, comprising 224,135 participants, were included. These examined DHIs targeting physical activity (7 reviews), dietary habits (3 reviews), alcohol consumption (2 reviews), combined alcohol and nicotine use (1 review), and obesity management (1 review), with an additional 6 reviews covering multiple health behaviors. Across reviews, 65% (13/20) reported statistically significant positive effects on at least one health behavior outcome. “Social support (unspecified)” was the most consistently adopted and effective BCT, especially with parental/peer involvement. The combination of “self-monitoring,” “goal setting,” and “feedback” also commonly appeared in successful interventions. Intervention effectiveness appeared linked to strategic BCT selection and individualization rather than the total number of techniques. The methodological quality of included reviews was predominantly low, with only 2 rated high.

**Conclusions:**

This umbrella review identified “social support (unspecified)” as a consistently effective BCT across multiple adolescent health behavior domains, particularly with parental/peer involvement. Intervention success appears linked to targeted and individualized BCT use. Future research should prioritize clarifying the specific components and delivery methods of effective social support, rigorously evaluating BCT configurations in underexplored areas such as adolescent smoking cessation, and examining their long-term impact on behavior change.

## Introduction

Adolescence is a critical period for the development of health behaviors that may persist into adulthood. Research indicates that early adolescence (10‐14 years old) is often characterized by experimentation and the formation of new habits, while late adolescence (15‐19 years old) sees an increased likelihood of risk-taking behaviors [1]. During this developmental period, many young people engage in behaviors that can negatively affect their immediate and long-term health outcomes, including poor dietary habits, insufficient physical activity, initiation of alcohol use, and smoking [2]. The prevalence of these risk behaviors among adolescents has led to increasing concern about their potential contribution to the development of noncommunicable diseases in later life [3].

Digital health interventions (DHIs) have emerged as a promising tool for promoting positive behavior change among adolescents. Defined by the World Health Organization as the discrete functionality of digital technology that is applied to achieve health objectives [4] and characterized in public health research as complex interventions [5], DHIs encompass a diverse array of technical formats, including mobile health apps, web-based programs, sensor-based systems, and telehealth services, to deliver health-related content and support, offering benefits such as increased accessibility, scalability, and the potential for real-time monitoring and personalized feedback [6].

The effectiveness of DHI has been demonstrated in various health behavior domains, with studies showing positive outcomes in improving alcohol consumption patterns, nicotine use behaviors, physical activity levels, dietary habits, and weight management among adolescents [2,7].

Behavior change techniques (BCTs) are the active ingredients of DHI, representing theory-based methods designed to alter the psychological determinants of behavior and facilitate sustained behavioral modification [8,9]. Grounded in established psychological theories such as social cognitive theory [10], the theory of planned behavior [11], the COM-B (capability, opportunity, motivation–behavior) model [12], and self-determination theory [13], BCTs operationalize theoretical constructs into specific, implementable intervention components. These techniques are designed to target key mechanisms of behavior change, which have been synthesized within frameworks like the COM-B model as capability, opportunity, and motivation [8,14]. The systematic identification and classification of BCTs has evolved considerably and ultimately refined into the comprehensive 93-technique taxonomy, the Behavior Change Technique Taxonomy version 1 (BCTTv1), which provides the current gold standard for BCT identification and reporting [9]. BCTTv1 offers standardized labels and detailed definitions for 93 distinct BCTs, hierarchically organized into 16 broader categories, designed to be used across behavioral domains and theoretical approaches.

Within DHI targeting adolescents, commonly deployed BCTs include “goal setting (behavior),” “self-monitoring of behavior,” “social support (unspecified),” and “feedback on behavior” [15], though the effectiveness of these techniques varies considerably across health behavior domains and implementation contexts. Meta-analytic evidence demonstrates that interventions incorporating multiple, theoretically coherent BCTs typically achieve superior behavioral outcomes compared to single-technique approaches, yet significant gaps remain in understanding optimal BCT combinations for specific adolescent populations and health behaviors [16,17].

Despite the growing body of evidence supporting DHI for adolescent health promotion, significant methodological and reporting deficiencies have limited the field’s capacity to understand which BCTs drive intervention effectiveness. While systematic reviews have examined DHI targeting specific adolescent health behaviors, including dietary modification, obesity prevention, physical activity promotion, alcohol consumption reduction, and smoking cessation [2,6,15,18,19], these reviews have revealed concerning gaps in BCT identification and reporting. Notably, Champion et al [19] found that few studies of school-based eHealth interventions described specific BCTs, and critically, none referenced established BCT taxonomies, severely limiting replication potential and theoretical advancement. This pattern of inadequate BCT reporting extends across multiple health domains and represents a fundamental barrier to developing evidence-based intervention protocols. While efforts to systematically examine BCTs have been undertaken in adult populations, yielding valuable insights for intervention optimization [20,21], to our knowledge, no comprehensive synthesis has examined BCT deployment and effectiveness specifically within adolescent DHI for adolescent health promotion. Such a synthesis is essential to identify which techniques demonstrate consistent effectiveness across different health behaviors, determine whether universal BCTs exist for adolescent populations, and establish evidence-based guidelines for intervention developers. Furthermore, a systematic examination of BCT effectiveness patterns would facilitate replication of successful interventions, inform the development of more potent multibehavioral interventions, and guide implementation research by identifying the most promising techniques for specific adolescent health outcomes.

Furthermore, recent technological advances and the increasing digitalization of health services have led to a rapid evolution in the design and delivery of DHI for adolescents [22]. This rapid advancement, coupled with inconsistent reporting of BCTs across studies, urges the need for a systematic umbrella review to synthesize and assess the current evidence base on the effectiveness of different BCTs in DHI targeting adolescent health behaviors.

Therefore, this systematic umbrella review aims to (1) identify and summarize evidence on the effectiveness of BCTs used in DHI targeting adolescents’ alcohol consumption, nicotine use, physical activity, dietary habits, and obesity management and (2) assess the strength of evidence for different BCTs across different digital solutions and health behaviors. By consolidating and analyzing the existing evidence base, this review will provide valuable insights for researchers, health care providers, and intervention developers working to improve adolescent health outcomes through DHI.

## Methods

### Identification of Studies

This systematic umbrella review was not prospectively registered; however, the review protocol adhered to established umbrella review methodologies and followed the PRIOR (Preferred Reporting Items for Overviews of Reviews) reporting guidelines [23]. A comprehensive literature search was conducted across 4 electronic databases: PubMed, PsycInfo, Embase, and CINAHL in April 2024, with no date restrictions. The search strategy used combinations of relevant keywords and indexed terms covering health domains and DHI. We did not apply any language restrictions. However, gray literature, including conference proceedings, dissertations, and unpublished reports, was excluded. While our inclusion criteria encompassed internet-based, computer-based, and mobile/phone-based interventions, we use the term DHI throughout this review for conciseness. The initial screening process involved reviewing titles and abstracts, followed by a detailed assessment of full-text articles for studies that potentially met our inclusion criteria. Two researchers from our team performed the search and screening processes independently. Any discrepancies were resolved through discussion. A third researcher was consulted to provide an ultimate decision when consensus could not be reached. The complete search strategy used for PubMed can be found in Section B of Multimedia Appendix 1.

### Eligibility Criteria

Inclusion criteria specified that eligible reviews must involve adolescent participants between 10 and 19 years of age; address either preventive measures or treatment approaches; examine health promotion interventions targeting specific behavioral indicators within one or more key domains, including alcohol consumption (frequency, quantity, and binge drinking patterns), nicotine use encompassing both traditional smoking and vaping behaviors (initiation, cessation, and reduction), physical activity (moderate to vigorous physical activity, sedentary behavior, and structured exercise), dietary habits (fruit and vegetable intake, sugar-sweetened beverage consumption, fast food consumption, and overall dietary quality), or obesity management and prevention (weight status, body composition, and eating behaviors); and provide either quantitative or qualitative data regarding BCTs. To ensure the highest level of evidence synthesis, we excluded non–systematic reviews and reviews that did not report a systematic search strategy.

Given the anticipated scarcity of eligible reviews focusing exclusively on adolescents, we included reviews with broader age ranges provided that they included studies specific to the adolescent participants that could be clearly isolated and summarized independently from other studies that focused on other age groups. Therefore, in all cases, only findings specific to adolescents were extracted. For reviews with slightly broader age eligibility criteria, inclusion decisions were based on the mean ages reported for the individual primary studies within those reviews. While this approach ensured that the central tendency of the samples included fell within the adolescent range, it remains possible that some of the primary studies included in these systematic reviews had a proportion of participants slightly outside of our 10‐19-year criterion. We excluded studies that targeted adolescent behavior change indirectly through parent, teacher, or health care provider intermediaries rather than engaging with adolescents as the primary intervention recipients.

### Data Synthesis

Our approach to evidence synthesis and classification was based on the methodological frameworks established by Michie et al [17] and Mair et al [3]. To ensure consistency, BCTs reported within the included reviews were systematically mapped to BCTTv1 [9]. Where source reviews used earlier taxonomies [8,24], BCTs were translated to their BCTTv1 equivalents by the review authors, referencing established mapping resources where available. BCT identification and coding were conducted independently by 2 researchers (NB and PdR). The coding was primarily based on the BCT descriptions and labels reported in the included reviews, rather than in the original primary studies. Any discrepancies between the coders were resolved through discussion. A third researcher was available to resolve any disagreements that could not be settled by the other two coders. No formal coding manual beyond the BCTTv1 taxonomy definitions and the established mapping resources for earlier taxonomies was used. A complete list of all BCTs identified in this review, alongside their BCTTv1 definitions, is provided in Section A of Multimedia Appendix 1.

To assess the evidence of BCTs, we implemented a systematic classification approach inspired by the Mair framework [3], which aimed to portray the evidence of the identified BCTs and their respective outcomes. The evidence appraisal process followed a structured approach that emphasized quantitative over qualitative findings. Meta-regression and subgroup analyses were assigned greater weight due to their ability to provide more robust conclusions on association or causality. In contrast, descriptive analyses and narrative evaluations were of lower evidential value. Although these qualitative approaches provide rich contextual understanding and insights into processes, they often struggle to establish direct causality. To provide a comprehensive overview of our findings, we created an effectiveness table (Multimedia Appendix 2) based on the Mair framework. This table synthesizes the results of all the reviews analyzed and provides a clear visualization of the evidence for each encountered BCT across different health behavior domains.

In addition, we carefully assessed the demographics of participants in individual studies within these reviews. To maintain consistency, we excluded evidence from studies that reported mean participant ages outside our predefined criteria, as well as those that did not report mean age.

### Assessment of Study Quality

The methodological quality of the included reviews was assessed using AMSTAR-2 (A Measurement Tool to Assess Systematic Reviews 2) [25]. The AMSTAR-2 framework includes 16 different evaluation criteria, with responses categorized as “yes,” “partly yes,” or “no.” The overall quality rating follows a tiered classification system: a study receives a “high” quality rating if no more than one noncritical criterion is not met, a “medium” rating if multiple noncritical criteria are not met, a “low” quality rating if a single critical criterion is not met, and a “very low” quality rating if multiple critical criteria are not met.

## Results

### Selection and Inclusion of Studies

**Figure 1.**
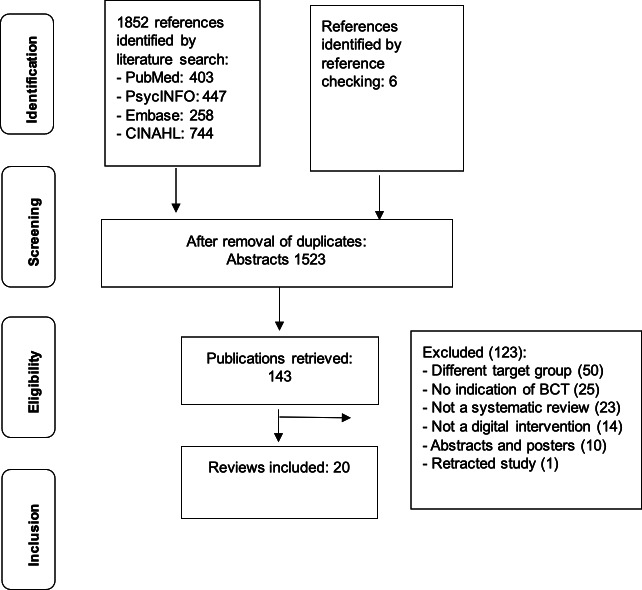
Flowchart depicting the study selection process. BCT: behavior change technique.

Our systematic search yielded 1852 initial references, which were reduced to 1523 unique studies after removal of duplicates. Initial screening of titles and abstracts identified 143 potentially eligible publications for detailed full-text assessment. We excluded 122 publications for a variety of reasons such as not being review articles, addressing populations outside our target population, lacking BCT documentation, not focusing on DHI, being conference abstracts or poster presentations, and addressing health behaviors beyond our scope of interest. The final analysis included 20 reviews representing a range of methodological approaches: 12 systematic reviews, 6 systematic reviews with meta-analysis, 1 systematic umbrella review, and 1 systematic scoping review. The flowchart detailing the inclusion process is presented in Figure 1.

### Study Quality

Our quality assessment using the AMSTAR-2 tool revealed considerable variation in the methodological quality of the 20 included reviews. The assessment identified only 2 reviews that were rated with high quality [26,27]. Most of the reviews had methodological limitations, with 10 of them rated as low quality [2,6,15,28-34] and 8 as critically low quality [35-42]. Common shortcomings included a lack of preregistration of the protocol, inadequate reporting of excluded studies, a lack of transparency of funding sources, and a lack of 2 independent team members to perform screening and data extraction. For an overview of all study quality ratings according to the AMSTAR-2 tool, created with the amstar2Vis R package [43], readers are referred to Section C of Multimedia Appendix 1. Moreover, given the possibility of overlap between primary studies in the included systematic reviews, we decided to map this overlap to indicate how it might affect the classifications of the BCTs in terms of their effectiveness. For a detailed overview, please see Section D of Multimedia Appendix 1.

### Health Behavior Domains

#### Overview of Synthesis Approach

The following sections detail the findings from the 20 included reviews, organized by the primary health behaviors targeted by the interventions within those reviews. Readers seeking a structured overview of the presence and effectiveness ratings of BCTs across all systematic reviews and health behavior domains should consult Multimedia Appendix 2. The narrative subsections below provide the interpretive context and supporting evidence for those classifications. This organizational approach was chosen because the included source reviews predominantly focused their analyses and BCT reporting within specific health behavioral contexts. Synthesizing evidence per domain allows for a nuanced understanding of how BCTs are applied and their reported effectiveness in relation to distinct adolescent health challenges. Throughout the subsequent presentation of findings, BCTs will be referred to using standardized codes or labels from the BCTTv1 taxonomy [9]. We acknowledge that these specific identifiers may not be immediately familiar to all readers. As it is not feasible to describe the meaning of all identified BCTs within the main text, readers are directed to Section A of Multimedia Appendix 1 for a detailed description of each BCT discussed in this review.

#### Physical Activity

##### Review Characteristics

Physical activity in the context of the reviewed interventions typically encompassed behaviors such as increasing moderate to vigorous physical activity, reducing sedentary time, or engaging in structured exercise.

Our analysis of physical activity interventions encompassed 7 reviews, collectively representing 14,593 adolescent participants. These included 6 systematic reviews by Carlin et al [28] (3656 participants), Baumann et al [29] (1515 participants), Seims et al [26] (1182 participants), Lau et al [35] (1456 participants), Liang et al [30] (2517 participants), and Creaser et al [31] (1843 participants), and 1 systematic scoping review by Lee et al [36] (2424 participants). Among these, the review by Baumann et al [29] incorporated a meta-analytic component.

##### Synthesis of BCTs in Physical Activity Interventions

Across the 7 reviews, several BCTs were consistently reported, though evidence for their independent effectiveness varied. “Social support (unspecified)” emerged as the most prevalent, appearing in all 7 reviews. Two reviews provided narrative syntheses indicating its positive effectiveness (O+) [26,28]. Specifically, Seims et al [26] highlighted enhanced social support via telehealth coaching and group participation as primary contributors to success in a trial, noting higher attrition in single-player versus multiplayer game versions and benefits from mentors and peers for adolescent girls in online sessions. Carlin et al [28] also identified “social support” as an effective component in successful interventions. Conversely, Creaser et al [31] reported mixed or inconclusive effects for this BCT, while the remaining reviews noted its presence without a formal effectiveness assessment [29,30,35,36].

“Self-monitoring of behavior” was also documented in all 7 reviews, although evidence for its effectiveness was less definitive. Two reviews reported mixed or inconclusive evidence (Ø) regarding its impact [28,31]. Carlin et al [28] suggested that pedometer-based “self-monitoring of behavior” showed promise when combined with “adding objects to the environment” and informational sessions [28]. Five other reviews primarily documented its presence or frequency without specific effectiveness analysis [26,29,30,35,36]; Lau et al [35] and Creaser et al [31] identified it as one of the most frequently implemented BCTs.

“Feedback on behavior” was investigated in 6 reviews [26,28,29,31,35,36]. Available analyses suggested mixed or inconclusive evidence (Ø) for its effectiveness [28,31]. Carlin et al [28] included it in their “feedback and monitoring” category, which appeared in successful interventions but also in trials with unfavorable follow-up results. Seims et al [26] noted the consistent presence of this BCT in effective interventions; however, they could not establish direct causality.

“Goal setting (behavior)” was reported in 6 reviews [26,28,30,31,35,36]. Carlin et al [28] classified interventions with “goal setting (behavior)” as having mixed evidence (Ø), as BCTs from their “goals and planning” category were present in both effective interventions and those with unfavorable follow-up results. However, they also noted that successful interventions predominantly combined “feedback and monitoring” with “goals and planning” categories. Lau et al [35] and Creaser et al [31] identified it as among the most frequently implemented BCTs, and Baumann et al [29] highlighted “goal setting (behavior)” as the primary technique used for personalizing interventions.

Finally, “adding objects to the environment” was investigated in 5 reviews [26,28,30,31,36]. Carlin et al [28] reported positive outcomes based on lower quality evidence (O+), while Creaser et al [31] indicated mixed results (Ø). Seims et al [26] noted that “adding objects to the environment” (specifically active video game equipment and DVDs) was uniquely present across all effective interventions they reviewed and frequently co-occurred with “goal setting (behavior)” and “feedback on behavior,” though direct causality was not established. Creaser et al [31] identified it (particularly wearable devices) as one of the most frequently implemented BCTs.

##### Key Findings From Reviews

Beyond individual BCTs, several broader themes emerged from the analysis of physical activity interventions. One significant theme concerned the relationship between the number of BCTs and intervention effectiveness. Evidence here was conflicting: Creaser et al [31] found no significant correlation between effectiveness and the quantity of BCTs used, whereas Seims et al [26] observed that while their most effective intervention incorporated multiple BCTs, the study with the highest total number of BCTs showed no effectiveness, suggesting strategic selection may be more critical than sheer quantity. Lau et al [35] reported that individual studies typically incorporated between 3 and 9 techniques.

Intervention characteristics and engagement also appeared influential. Creaser et al [31] suggested that success might depend more on engagement quality, such as frequency and duration of interaction, than on the mere presence or number of BCTs. Relatedly, Baumann et al [29] narrative synthesis suggested that gamified approaches could be effective when combining individualization, strong theoretical foundations, and integrated BCTs. The role of individualization was further explored in Baumann et al [29] meta-analysis, which demonstrated a small but significant overall effect of DHI on insufficient physical activity (Cohen *d*=0.33), whereas low-individualization interventions showed no effect, a statistically significant difference (*P*=.04) [29]. For sedentary behavior, however, no significant differences between low and high individualization were found [29]. As this review did not assess individual BCTs, their presence was noted (✓) in our effectiveness table (Multimedia Appendix 2) without evidence ratings.

The application and reporting of theory was another key theme. Lau et al [35] found that meaningful comparisons of behavior change theories’ effectiveness were hampered by a scarcity of theory-based interventions and heterogeneous study designs. Similarly, Baumann et al [29] noted that mobile health interventions rarely incorporate a theory-driven approach and that there is little emphasis on evidence-based content. Lastly, reporting and methodological limitations within the primary studies were highlighted by several reviews. Seims et al [26] pointed out that only 28% of BCTs from the BCTTv1 taxonomy were identified in their reviewed trials. The length of the interventions varied considerably across the physical activity systematic reviews and may have affected the effectiveness of the BCTs. For example, Lau et al [35] reported durations ranging from 4 weeks to 12 months, whereas Creaser et al [31] included studies spanning 8 to 52 weeks. As BCT effects, particularly those related to self-monitoring and goal setting, may require sustained exposure to generate and consolidate behavioral changes, the heterogeneity in intervention length complicates direct comparisons of effectiveness across studies.

### Dietary Habits

#### Review Characteristics

Interventions focused on dietary habits within the included reviews primarily aimed to promote increased consumption of fruits and vegetables, reduce the intake of sugar-sweetened beverages or unhealthy snacks, and improve overall dietary quality. Our synthesis for this domain is based on 3 reviews: 2 systematic reviews by Hsu et al [15] (3554 participants) and Vézina-Im et al [42] (152,001 participants), and 1 systematic umbrella review by Capper et al [37], which did not report aggregate participant numbers for its selected reviews. For the umbrella review by Capper et al [37], our BCT analysis specifically considered 4 of their included systematic reviews [44-47] that focused on DHI. It is noteworthy that none of these 4 reviews conducted statistical analyses to directly evaluate BCT effectiveness, providing only narrative descriptions.

#### Synthesis of BCTs in Dietary Interventions

The most commonly identified BCTs across these reviews included “goal setting (behavior),” “self-monitoring of behavior,” “social support (unspecified),” “feedback on behavior,” and “restructuring the physical environment.” Capper et al [37] identified 4 BCTs as effective (O+) based on their synthesis of narrative findings: “feedback on behavior,” “self-monitoring of behavior,” “social support (unspecified)” (particularly involving peers and parents), and “restructuring the physical environment” (especially when combined with educational elements). Hsu et al [15], who examined social media interventions for adolescent nutrition, documented the presence of several BCTs, noting that “social support (unspecified)” was present in all interventions, typically delivered via social media features. Other frequent BCTs in their review were “demonstration of the behavior,” “self-monitoring of behavior,” “goal setting (behavior),” and “feedback on behavior.” While Hsu et al [15] reported modest short-term improvements in adolescent nutrition behaviors and observed these BCTs in successful interventions, they emphasized the need for more research to confirm their effectiveness. The included studies neither provided statistical analyses of BCT effectiveness nor offered a narrative synthesis of how these techniques influenced behavior change. As a result, we could only record the presence of identified BCTs without effectiveness ratings (✓).

Vézina-Im et al [42] found it challenging to determine the effectiveness of individual BCTs because interventions typically used multiple techniques simultaneously. Their review, which used Cane et al [48] taxonomy, identified “information about health consequences” as the most prevalent BCT (reported in 72.2% of studies). Other common BCTs included “restructuring the physical environment” (47.2%), “goal setting (behavior)” (36.1%), “self-monitoring of behavior” (33.3%), and “social support (unspecified)” (30.6%).

#### Key Findings From Reviews

Several broader findings emerged. Capper et al [37] emphasized that DHI incorporating multiple, complementary approaches, such as combining educational elements with “restructuring the physical environment,” using “feedback on behavior,” involving peers and parents through “social support (unspecified),” and applying behavioral theory, demonstrated greater success rates. They also noted that online content and messaging showed positive dietary behavior changes, proving particularly engaging for adolescents in secondary schools. Vézina-Im et al [42] observed a distinction in BCT application based on intervention type: legislative or environmental interventions typically used a single BCT, most commonly “restructuring the physical environment,” whereas educational or behavioral interventions tended to use multiple BCTs. A significant limitation across this domain was the general absence of quantitative analyses evaluating the effectiveness of individual BCTs within the reviewed systematic reviews, which was often due to the co-occurrence of multiple BCTs or the absence of such analyses in the primary studies themselves. Furthermore, the length of interventions included in the dietary studies varied widely, from single-session exposures to multimonth programs, and was rarely analyzed as a moderator of BCT effectiveness. Given the role of intervention duration in shaping the consolidation of dietary habits, this represents an important gap in the current evidence base.

### Alcohol Consumption

#### Review Characteristics

Interventions targeting alcohol consumption among adolescents, as covered in the reviewed literature, primarily focused on aspects such as reducing the frequency and quantity of alcohol intake, preventing binge drinking episodes, and delaying the initiation of alcohol use. Our analysis of DHI for alcohol consumption is based on 3 reviews: a systematic review by Hutton et al [39], a meta-analysis by Smedslund et al [27], and a systematic review and meta-analysis by O’Logbon et al [33], which examined interventions targeting both alcohol and nicotine use; for the latter, we focus specifically on their findings related to alcohol consumption. The review by Hutton et al [39] provided qualitative descriptions of BCTs but did not include quantitative analyses of their specific effectiveness.

#### Synthesis of BCTs in Alcohol Interventions

The BCT “feedback on behavior,” particularly in the form of personalized feedback, was a central focus in the evidence for alcohol consumption. Smedslund et al [27] meta-analysis demonstrated that assessment and feedback interventions significantly reduced alcohol consumption compared to no-intervention controls, an effect evident both immediately postintervention (standardized mean difference [SMD] −0.17) and at long-term follow-up (SMD −0.17). O’Logbon et al [33] narrative synthesis further explored feedback approaches, highlighting personalized feedback and interactive monitoring. They cited Bryant et al [49], whose findings indicated that email-based personalized feedback effectively reduced weekly drinking and binge drinking. However, the synthesis also included Hides et al [50], who found that a behavioral monitoring and feedback app, while improving alcohol-related knowledge, did not significantly impact drinking behaviors. Consequently, due to such contradictory findings within the studies they reviewed, O’Logbon et al [33] classified “feedback on behavior” and associated “self-monitoring of behavior” techniques as having mixed evidence (Ø) in their overall synthesis for alcohol interventions. Beyond these, while other BCTs were identified within the studies included across the reviews, they were generally not subjected to dedicated statistical analyses or comprehensive narrative syntheses to evaluate their independent effectiveness.

#### Key Findings From Reviews

The overall effectiveness of DHI for alcohol reduction was supported by meta-analytic evidence. Smedslund et al [27] found that computerized assessment plus feedback interventions yielded comparable effect sizes in reducing alcohol consumption when compared against assessment-only interventions (SMD −0.15), though the distinct benefit of adding feedback was maintained only in the short term. Complementing this, the meta-analytic component of O’Logbon et al [33] review revealed modest but significant reductions in weekly alcohol consumption for DHI targeting alcohol use when compared to controls (SMD −0.12).

A significant limitation in this domain is that, apart from “feedback on behavior” and to some extent, “self-monitoring of behavior,” many BCTs were not evaluated individually for their effectiveness within the reviews. Furthermore, intervention duration was inconsistently reported and rarely examined as a moderator. Smedslund et al [27] observed that, although interventions involving assessment and feedback produced significant short-term reductions (SMD −0.17), the additional benefit of providing feedback in addition to assessment was not evident in the outcome at the long-term follow-up. This suggests that the dosage and duration of BCTs are important factors for interpreting their effectiveness in this domain.

### Obesity Management

#### Review Characteristics

Obesity management interventions in the reviewed literature aimed to address overweight and obesity primarily through changes in weight status, such as BMI or BMI *z* score, body composition, or associated energy balance-related behaviors, including diet and physical activity. Our synthesis for this domain is based on a single systematic review and meta-analysis by Azevedo et al [32]. This review incorporated 19 studies, encompassing 2352 overweight or obese children and adolescents under the age of 17 years. It is important to note that while Azevedo et al [32] conducted detailed subgroup analyses on various study characteristics, they did not specifically evaluate the independent effectiveness of individual BCTs through statistical means. Consequently, the assessment of BCT effectiveness presented here relies on their narrative analysis of the included studies.

#### Synthesis of BCTs in Obesity Interventions

Azevedo et al [32] identified 12 distinct BCTs across the interventions they examined: “goal setting (behavior),” “problem-solving,” “feedback on behavior,” “self-monitoring of behavior,” “social support (unspecified),” “instruction on how to perform the behavior,” “information about health consequences,” “demonstration of the behavior,” “prompts/cues,” “material incentive (behavior),” “nonspecific reward,” and “adding objects to the environment.” Based on their narrative synthesis, 6 of these identified BCTs showed evidence of being part of effective interventions: “goal setting (behavior),” “problem-solving,” “self-monitoring of behavior,” “social support (unspecified),” “adding objects to the environment,” and “nonspecific reward.” The review highlighted that 8 of the 19 included studies demonstrated significant reductions in BMI or BMI *z* score in intervention groups compared to controls. For instance, effective interventions included home-based active video games, such as the study by Staiano et al [51], which implemented “adding objects to the environment” (wearable activity trackers) combined with “social support (unspecified)” (video consultations with fitness coaches). Other successful approaches used online solutions; Johansson et al [52] used an app and website incorporating “nonspecific reward,” “self-monitoring of behavior,” and “goal setting (behavior),” while Ahmad et al [53] used “self-monitoring of behavior,” “goal setting (behavior),” and “problem-solving” via social media and other communication channels.

#### Key Findings From Reviews

Several broader findings emerged from Azevedo et al [32] review. According to their narrative synthesis, active video game interventions consistently demonstrated reductions in BMI or BMI *z* score, whereas other digital health approaches, such as those primarily using social media, showed more variable effectiveness. The meta-analysis conducted by Azevedo et al [32] revealed a modest but statistically significant advantage for DHI over control groups in reducing BMI or BMI *z* score (SMD −0.31, 95% CI −0.49 to −0.13; *P*<.001). This effectiveness was observed across different age groups, including participants under 12 years (SMD −0.183; *P*=.03) and those aged 12‐17 years (SMD −0.633; *P*=.03), as well as across varying intervention lengths, from 6 to 12 months (SMD −0.301; *P*=.02) to 12 months or more (SMD −0.342; *P*=.02), and was noted regardless of the primary studies’ methodological quality. Furthermore, their subgroup analyses indicated that interventions targeting 1 or 2 behaviors (either physical activity and/or dietary habits) led to significant improvements, while interventions designed to address 3 behaviors simultaneously (physical activity, sedentary behavior, and dietary habits) did not show significant effects. The review by Fleischman et al [54], included by Azevedo et al [32], also found that combining telehealth consultations with obesity specialists with in-person primary care visits was more effective at reducing BMI *z* score than primary care alone.

### Nicotine Use

#### Review Characteristics

Nicotine use interventions within the scope of this review targeted behaviors such as the prevention of smoking or vaping initiation, support for cessation efforts, or reduction in the use of tobacco and nicotine-containing products among adolescents. The findings for this health behavior domain were obtained from a systematic review and meta-analysis conducted by O’Logbon et al [33]. The review originally examined interventions targeting alcohol and nicotine use separately and included 37 studies with 14,330 participants who met the age criteria. The synthesis below focuses specifically on the outcomes related to nicotine use interventions, as reported by O’Logbon et al [33].

#### Synthesis of BCTs in Smoking Interventions

The narrative synthesis within O’Logbon et al [33] indicated that findings for interventions targeting nicotine use were relatively consistent for certain BCTs. Notably, compelling evidence was reported for “information about health consequences.” For instance, Mays et al [55], who developed a 6-week SMS text messaging intervention to highlight the short- and long-term health risks associated with hookah tobacco, including toxin exposure and addiction potential, found that 30% of participants achieved complete smoking cessation, while an additional 20% reduced their smoking frequency at 6 weeks postintervention. Consequently, “information about health consequences” was assigned a positive effectiveness rating (O+). Furthermore, O’Logbon et al [33] highlighted the effectiveness of online peer coaching, categorized under the BCT “social support (unspecified).” Positive outcomes were cited from An et al [56] smoking cessation trial, leading to a positive effectiveness rating (O+) for “social support (unspecified)” in the context of nicotine interventions.

#### Key Findings From Reviews

Despite the positive findings for specific BCTs like “information about health consequences” and “social support (unspecified)” identified within individual studies, the broader meta-analytic component of O’Logbon et al [33] review revealed a more nuanced picture of overall intervention effectiveness for nicotine use. Their meta-analysis showed no statistically significant effect of DHI on 30-day smoking abstinence rates when compared to controls. Additionally, it is noteworthy that the only nicotine use study with positive cessation outcomes [55] used a relatively brief 6-week SMS text messaging intervention, while the broader meta-analysis included studies of various durations. Given the absence of treatment duration as a moderator variable in the available reviews, it remains unclear what the optimal intensity and length of BCT delivery are for achieving positive cessation outcomes in adolescents.

### Multiple Health Behavior Domains

#### Review Characteristics

Beyond interventions targeting singular health behaviors, a significant portion of the reviewed literature examined DHI designed to simultaneously address 2 or more health domains. This approach recognizes the interconnectedness of many adolescent health behaviors (eg, diet and physical activity in relation to obesity) and the potential for multicomponent interventions to achieve broader lifestyle improvements. The following subsections synthesize findings from reviews that adopted such a multibehavioral focus, categorized by the specific combination of health domains targeted.

#### Physical Activity and Dietary Habits

##### Review Characteristics

Interventions targeting combined physical activity and dietary habits were examined in 2 reviews: a systematic review by Rose et al [6], which included approximately 2915 participants, and an analysis of mobile apps by Schoeppe et al [41], which focused exclusively on app content without involving study participants. Both reviews conducted a narrative synthesis of their findings. However, Schoeppe et al [41] limited their analysis to identifying the BCTs present in mobile apps, meaning that our understanding of intervention effectiveness for this combined domain is primarily derived from the narrative findings of Rose et al [6].

##### Synthesis of BCTs in Physical Activity and Dietary Interventions

According to Rose et al [6], 3 key BCTs were associated with successful behavior change: “goal setting (behavior),” “self-monitoring of behavior,” and “social support (unspecified),” particularly in the form of parental involvement. Their findings suggested that “goal setting (behavior)” was particularly effective, leading to meaningful improvements in dietary habits, physical activity, or both. Furthermore, “self-monitoring of behavior” reportedly yielded better results when combined with “goal setting (behavior)” rather than when used in isolation. Interventions that involved parents [“social support (unspecified)"] also typically achieved significant improvements in these health behaviors. In their broader narrative analysis, Rose et al [6] identified several techniques with positive outcomes: “goal setting (behavior),” “problem-solving,” “feedback on behavior,” “self-monitoring of behavior,” “social support (unspecified),” “social support (practical),” “instruction on how to perform the behavior” (which included dietary education), and “information about health consequences.”

Schoeppe et al [41], in their analysis of mobile apps, did not evaluate the effectiveness of BCTs but focused on their frequency. They identified 11 BCTs across the apps reviewed: “feedback on behavior,” “self-monitoring of behavior,” “social support (unspecified),” “instruction on how to perform the behavior,” “information about health consequences,” “social comparison,” “prompts/cues,” “material incentive (behavior),” “nonspecific reward,” “reward (outcome),” and “restructuring the physical environment.” Their analysis of 25 apps targeting young people (12 for dietary habits, 18 for physical activity, and 7 for sedentary behavior) revealed that apps typically contained an average of 6 BCTs, with a range from 1 to 14. The most commonly implemented techniques were “instruction on how to perform the behavior” (found in 19 apps), “social support (unspecified)” (18 apps), both “nonspecific reward” and “reward (outcome)” (17 apps each), and “feedback on behavior” (13 apps).

##### Key Findings From Reviews

A key finding from Rose et al [6] was the observation that while digital interventions could effectively improve adolescents’ dietary habits and physical activity behaviors, these improvements often did not persist over time. Schoeppe et al [41] reported a finding related to app characteristics, noting that apps with higher quality ratings tended to include more technical features (ρ=0.42; *P*<.05) and a greater number of BCTs (ρ=0.54; *P*<.01).

### Physical Activity, Dietary Habits, and Obesity Management

#### Review Characteristics

Two reviews investigated interventions targeting combinations of physical activity, dietary habits, and obesity management. These included 2 systematic reviews, one of which featured a meta-analysis and meta-regression on SMS text messaging for weight management [34], and another on digital weight management interventions [40].

#### Synthesis of BCTs in Physical Activity, Dietary, and Obesity Interventions

Across these reviews, the strongest evidence for BCT effectiveness came from Kouvari et al [40] analysis of parental involvement, which they coded as “social support (unspecified)” and “social support (practical).” Their meta-analysis revealed that significant BMI reductions were only achieved in interventions that included parents (SMD –0.39, 95% CI –0.59 to –0.18; *P*<.001), whereas interventions without parental involvement showed no significant effects compared to controls. In their narrative synthesis, Kouvari et al [40] also highlighted the importance of “self-monitoring of behavior,” suggesting it may be an essential component for success as it was a key feature across the effective DHIs they reviewed.

The other reviews provided descriptive data on BCT implementation. Siopis et al [34] did not evaluate individual BCT effectiveness in their meta-regression. However, the primary studies they included offer some insight. For instance, an effective intervention by Napolitano et al [57] used a combination of “feedback on behavior,” “social support (unspecified),” and “self-monitoring of behavior,” while a nonsignificant intervention by Lubans et al [58] used “self-monitoring of behavior,” “adding objects to the environment,” and “social support (practical).” Due to the design of the identified reviews, we noted the presence of these BCTs without being able to assign effectiveness ratings (✓).

Collectively, “goal setting (behavior),” “self-monitoring of behavior,” and “social support (unspecified)” were the most commonly identified BCTs across the 3 reviews that examined this combined health domain.

#### Key Findings From Reviews

The broader meta-analytic findings from Kouvari et al [40] showed that technology-based interventions produced significantly greater BMI reductions compared to control groups (SMD –0.61, 95% CI –1.10 to –0.13; *P*=.01). This effect was most pronounced at the 6-month follow-up (SMD –0.37, 95% CI –0.72 to –0.03; *P*=.03).

The findings reported by Siopis et al [34] highlight the nuances of intervention design. In their review, the study by Napolitano et al [57] found that a combined approach using Facebook plus daily SMS text messages about “self-monitoring of behavior” goals led to significant weight loss (mean −2.4, SD 2.5 kg), whereas a Facebook-only approach did not. In contrast, the multicomponent intervention by Lubans et al [58], which included workshops, pedometers, and parent newsletters, found no significant difference in BMI compared to controls after 12 months.

Overall, strong quantitative evidence from one review [40] supports the effectiveness of “social support (unspecified)” and “social support (practical),” specifically through parental involvement. Promising qualitative evidence from the same review suggests “self-monitoring of behavior” is also a key component of effective interventions in this domain.

### Alcohol Consumption, Nicotine Use, Physical Activity, and Dietary Habits

#### Review Characteristics

Our synthesis for interventions addressing a combination of alcohol consumption, nicotine use, physical activity, and dietary habits is drawn from a systematic review by de Sousa et al [2]. This review, focused on DHI for adolescents, examines 14 studies that included 7616 participants. The review encompassed young people between 10 and 24 years old, with all reported mean ages falling within our study’s defined scope. Given the diversity of health behaviors targeted and substantial differences in study designs and outcomes, de Sousa et al [2] conducted a qualitative analysis, noting that the limited number of studies per specific health behavior and technique rendered quantitative analysis unfeasible.

#### Synthesis of BCTs in Alcohol, Smoking, Physical Activity, and Dietary Interventions

For the identification of BCTs, de Sousa et al [2] used Michie et al [9] taxonomy. Their review documented the presence of 8 specific BCTs within the interventions: “goal setting (behavior),” “feedback on behavior,” “self-monitoring of behavior,” “social support (unspecified),” “information about health consequences,” “prompts/cues,” “graded tasks,” and “adding objects to the environment.” It is important to note, however, that while de Sousa et al [2] identified these BCTs, their focus was primarily on the theoretical foundations of the interventions rather than on evaluating the effectiveness of individual BCTs.

#### Key Findings From Reviews

Key findings from de Sousa et al [2] review indicated that the results of the included interventions were largely positive, with 13 out of the 14 studies demonstrating meaningful improvements across the targeted health domains. These improvements included better physical activity levels and motivation, enhanced fitness and cardiovascular health markers, decreased problematic drinking and its associated consequences, reduced smoking intention among nonsmokers, and higher consumption of fruits and vegetables. A central observation was that all interventions included in their review were theory-based, using either single or combined theoretical frameworks. Single-theory approaches identified were the transtheoretical model/stages of change, operant conditioning theory, the information-motivation-behavioral skills model, and self-efficacy theory (derived from social cognitive theory). Some studies also integrated multiple theories, such as combinations of social cognitive theory with the transtheoretical model, or the theory of planned behavior with the health action process approach.

### Nicotine Use, Physical Activity, and Dietary Habits

#### Review Characteristics

Interventions addressing a combination of nicotine use, physical activity, and dietary habits were examined through one review by Edwards et al [38], which focused on mobile apps. This review analyzed 64 mobile apps designed to target multiple health behaviors, with a majority focusing on exercise promotion (70%), followed by fitness improvement (17%), smoking cessation (6%), and other areas such as oral hygiene, weight management, and blood glucose monitoring adherence. The primary aim of Edwards et al [38] was to examine health apps incorporating gaming elements and to analyze their BCTs; however, their investigation did not extend to evaluating how individual BCTs affected user outcomes, focusing instead on the presence and combination of BCTs and their relationship with app characteristics like user ratings and pricing.

#### Synthesis of BCTs in Nicotine Use, Physical Activity, and Dietary Interventions

The analysis by Edwards et al [38] did not assess the effectiveness of BCTs but provided a detailed account of their prevalence within the reviewed mobile apps. Three predominant BCT groupings were identified: “feedback and monitoring” (present in 94% of apps), “comparison of behavior” (81%), and “reward and threat” (81%). Among specific techniques, 5 appeared most frequently: “self-monitoring of behavior” (86%), “nonspecific reward” (82%), “material incentive (behavior)” (82%), “social support (unspecified)” (75%), and “focus on past success” (73%). The review also highlighted common combinations of BCTs, noting that “goal setting (behavior)” was often paired with “self-monitoring of behavior,” “nonspecific reward,” and “material incentive (behavior)” (found in 55% of apps), or with “self-monitoring of behavior” and “focus on past success” (52% of apps).

#### Key Findings From Reviews

Several broader findings emerged from Edwards et al [38] examination of these multibehavior apps. It was observed that apps typically incorporated an average of 14 BCTs, with the number ranging from 5 to 22 per app. A notable observation was the underuse of the broader BCTTv1 taxonomy, as 42 of the 93 documented techniques were not present in any of the analyzed apps. Furthermore, when exploring app characteristics, the researchers found no significant correlation between the number of BCTs an app contained and its price (*P*=.45; *r*=0.10) for the 36% of apps that were paid applications.

## Discussion

### Principal Findings

This systematic umbrella review synthesizes evidence from 20 reviews, encompassing 224,135 adolescents aged 10‐19 years, to examine the effectiveness of DHIs and their incorporated BCTs across health behaviors including alcohol consumption, nicotine use, physical activity, dietary habits, and obesity management. A primary finding is the widespread implementation of “social support (unspecified),” which was reported in all included reviews, underscoring its perceived importance in interventions for young people. Our synthesis has identified clear patterns in the use and reported effectiveness of various BCTs, with some evidence supporting specific techniques and their combinations, particularly in interventions targeting physical activity, obesity management, and alcohol consumption. Conversely, the evidence base for BCTs in dietary and nicotine use interventions appeared more heterogeneous.

The widespread reporting of “social support (unspecified)” across all the interventions reviewed indeed suggests that it is perceived as being central to facilitating health behavior change among adolescents. However, the frequent “unspecified” classification raises complex issues. While it may, in some instances, reflect a lack of taxonomic precision in the primary studies or source reviews, it could also signify the deliberate use of complex support strategies that cannot be easily categorized, or interventions designed to be more ecologically valid than strictly standardized. Consequently, this pattern may not merely represent a methodological limitation in detail; it may also indicate that effective DHIs for adolescents often require flexible, context-responsive support mechanisms that can adapt to different individual needs.

A key finding across health domains was the consistent effectiveness of social support mechanisms, particularly those incorporating parental or peer engagement, a pattern that aligns with the meta-analytic evidence demonstrating significant positive effects of parent-based interventions on multiple adolescent lifestyle risk behaviors [59]. This was especially evident in obesity management interventions, where parental involvement significantly enhanced outcomes, while interventions without parental support showed no significant effects. Similarly, in physical activity interventions, social support emerged as both the most prevalent and effective technique, appearing across all 7 included reviews. However, an important observation was the relative absence of practical social support, identified in only 3 reviews, which may be attributed to the challenges faced by distinguishing between different types of social support outlined in Michie et al [9] taxonomy.

Digital platforms offer a variety of ways to provide support. Peer engagement could be encouraged through moderated forums, curated group chats, or anonymous peer-matching systems. Parental involvement could include access to dedicated portals providing guidance, progress updates, or tools to facilitate supportive conversations at home. Connections with teachers or health coaches could be maintained via secure messaging, scheduled video check-ins, or shared progress dashboards. It is crucial to distinguish between emotional, practical, informational, and appraisal support, as outlined in established frameworks, and to understand their differential impacts. The relative scarcity of “social support (practical)” in the reviewed literature, which was identified in only 3 reviews, may reflect the challenges of consistently applying detailed BCT taxonomies, such as BCTTv1, rather than an actual absence of such support in interventions.

Beyond social support, the combination of “self-monitoring of behavior,” “goal setting (behavior),” and “feedback on behavior” emerged as frequently implemented strategies, often associated with positive outcomes. This was particularly evident in interventions for alcohol consumption, where assessment and feedback approaches demonstrated significant effects. This combination of BCTs aligns with the foundational principles of control theory [60], which states that behavioral regulation is achieved through an iterative process of setting goals, tracking behavior, receiving feedback on performance in relation to these goals, and subsequently adjusting efforts or goals. The effectiveness of these BCTs when implemented together highlights the importance of equipping adolescents with self-regulation skills.

Interestingly, this review supports findings such as those by Seims et al [26] relating to physical activity interventions. Specifically, it suggests that the success of an intervention may not be directly correlated with the number of BCTs used. Interventions incorporating a wide range of techniques did not necessarily outperform those with a smaller, more carefully chosen set of BCTs. This emphasizes the potential importance of intervention coherence and carefully selecting BCTs that are theoretically matched to the target behavior and population, rather than taking a “more is better” approach.

### Implications for Practice and Future Research

A critical issue that requires further consideration is the assumption in much of this literature that BCTs, which were primarily developed and validated in face-to-face or experimental contexts, are similarly effective when implemented in digital formats [16,22]. However, there is growing evidence that the mode of delivery is not merely a vehicle but an active moderator of BCT efficacy [61,62]. This concern is particularly relevant in the case of “social support (unspecified),” the most prevalent BCT across our review. Face-to-face social support is characterized by reciprocity, emotional attunement, and relational continuity, features that are difficult to replicate through asynchronous messaging, automated prompts, or algorithmically mediated peer interactions [63]. Therefore, it is currently impossible to conclude that the high prevalence of this BCT in DHI equates to the effective delivery of the psychological construct it represents. The predominantly “unspecified” classification further obscures whether interventions leveraged emotional, informational, practical, or appraisal support, dimensions with distinct mechanisms of action [64]. Future research should apply more precise operationalizations of social support within DHI, distinguish between human-delivered and system-generated support, and directly assess whether digitally mediated social support activates the same psychological pathways as face-to-face support. Otherwise, effect estimates attributed to “social support (unspecified)” in DHI may substantially overstate or distort the role of this technique in producing behavioral change in adolescents.

Furthermore, the principle of individualization appears critical. Baumann et al [29] meta-analytic findings for physical activity interventions demonstrated superior outcomes for highly individualized approaches compared to those with low individualization. This strongly suggests that tailoring intervention content and BCT delivery to the specific needs, preferences, and contexts of individual adolescents is key to effectiveness.

Although this review primarily categorized interventions by their constituent BCTs, it is important to acknowledge that the effectiveness of these techniques is intrinsically linked to the quality and sophistication of the DHI itself. DHI characteristics, such as the use of sensor-based adaptations, the sequencing of content availability, and the degree of system-driven personalization, act as moderators of BCT efficacy. For example, a self-monitoring BCT may function differently within a static web-based diary than within a sensor-based app that provides adaptive feedback in real time. Therefore, future research must aim to disentangle the specific contribution of the BCT from the technological format and implementation quality in order to better understand these mechanisms.

Future research should extend this principle by systematically examining how BCT effectiveness may vary by key demographic factors such as sex and age, as such analyses are crucial for developing equitable and maximally impactful interventions.

### Limitations

The methodological quality of the included systematic reviews varied considerably. The majority were rated as low or critically low quality according to AMSTAR-2 criteria. It is important to note that AMSTAR-2 ratings were not formally weighted within the BCT effectiveness classifications presented in Multimedia Appendix 2 or the domain-specific narrative syntheses. All reviews contributed to the effectiveness ratings based on their reported findings, regardless of their methodological quality. The methodological quality of each included systematic review is reported separately in Section C of Multimedia Appendix 1, and we therefore encourage our readers to consult these ratings when interpreting any individual BCT effectiveness classification.

Limitations such as the lack of preregistered protocols and inadequate reporting on primary study selection and characteristics prevent definitive synthesis and interpretation. The potential for primary studies to overlap across the included reviews due to nonmutually exclusive eligibility criteria could also influence the independence and weighting of some findings. Furthermore, considerable heterogeneity in the methods used by the reviews for data synthesis and BCT identification complicated our comparative analysis. A related and underappreciated source of heterogeneity was intervention duration. It is reasonable to assume that BCT effectiveness depends on exposure length. Brief interventions may be sufficient to initiate behavior change, but sustained BCT delivery, particularly for techniques targeting self-regulation and social support, may be necessary for long-term behavior maintenance [16,17]. Across the included reviews, intervention duration ranged from single sessions to over 12 months, yet this variable was rarely analyzed as a moderator of BCT-specific effects. Future systematic reviews and primary studies therefore need to address this gap by systematically reporting intervention duration and, where feasible, incorporating it as a moderator in meta-analytic models. Heterogeneity in BCT reporting across primary studies and their subsequent reviews remains a fundamental barrier to drawing robust conclusions about the effectiveness of specific BCTs for different health behaviors and DHI.

### Conclusions

This systematic umbrella review represents a comprehensive synthesis of the current evidence on the effectiveness of BCTs within DHI targeting multiple health behaviors in adolescents. Our analysis indicates that “social support (unspecified)” is a consistently implemented BCT across various health domains, with particular promise noted when it involves parents or peers. Furthermore, interventions integrating “self-monitoring of behavior,” “goal setting (behavior),” and “feedback on behavior” appear frequently and align with established theoretical models of behavior regulation, showing utility in several health domains.

Despite these insights, our review has displayed significant gaps and inconsistencies in the literature. Interventions targeting nicotine use and alcohol consumption in adolescents, while reviewed, require more nuanced investigation, particularly concerning the long-term sustainability of effects and the specific BCTs or combinations thereof that drive positive outcomes. The evidence for BCT effectiveness in dietary interventions also remains notably heterogeneous. The findings strongly suggest that intervention effectiveness is more dependent on the strategic and theoretically informed selection and operationalization of BCTs, along with a high degree of individualization, rather than merely the total number of techniques included.

To advance the field of adolescent digital health promotion, several recommendations are proposed. First, researchers conducting primary studies should adopt standardized BCT reporting using established taxonomies, principally the BCTTv1. This includes detailed documentation of each BCT implemented, precise descriptions of how each BCT was operationalized within the DHI, and a clear articulation of any intended BCT combinations and their hypothesized synergistic or additive effects. Such methodological rigor will enhance the replicability of successful interventions and facilitate more robust evidence synthesis.

Second, since modern DHIs are often dynamic, using algorithms for personalization and sensor-based adaptation, evaluating them as fixed “treatment packages” via traditional 2-arm randomized controlled trials is insufficient. To address this methodological challenge, future research should prioritize optimization frameworks such as the multiphase optimization strategy or factorial experimental designs. These methodologies enable researchers to systematically dismantle complex interventions and isolate the specific contribution of distinct BCTs and delivery formats. This approach is essential for understanding how variable intervention components interact to drive effectiveness in heterogeneous adolescent populations.

Third, greater emphasis should be placed on exploring the mechanisms of action through which BCTs exert their effects, including the investigation of different types and sources of social support (eg, peer, parental, and professional) and their optimal integration into digital formats. Intervention developers and practitioners should leverage the existing evidence by prioritizing the inclusion of BCTs with demonstrated promise, such as tailored social support mechanisms and integrated self-regulatory BCT components, while co-designing interventions with adolescents to ensure engagement and relevance. Finally, systematic reviewers are urged to adhere to high-quality reporting standards and preregister protocols to improve the reliability of future evidence syntheses.

## Supplementary material

10.2196/84754 Multimedia Appendix 1 Behavior Change Technique Taxonomy version 1 definitions, PubMed search strategy, AMSTAR-2 quality assessment summary, and primary study overlap across included systematic reviews.

10.2196/84754 Multimedia Appendix 2 Summary of the effectiveness of health-related behavior change techniques across health domains.

10.2196/84754 Checklist 1 PRIOR checklist.

10.2196/84754 Checklist 2 PRISMA (Preferred Reporting Items for Systematic Reviews and Meta-Analyses) Checklist.

## Abbreviations

AMSTAR-2: A Measurement Tool to Assess Systematic Reviews 2
BCT: behavior change technique
BCTTv1: Behavior Change Technique Taxonomy version 1
COM-B: capability, opportunity, motivation–behavior model
DHI: digital health intervention
PRIOR: Preferred Reporting Items for Overviews of Reviews
SMD: standardized mean difference
