# Supplementary material for: Behavior Change Techniques in Digital Health Interventions for Promoting Adolescent Health Behaviors: Systematic Umbrella Review
Source: JMIR Ment Health. 2026 May 6;13:e84754. doi: 10.2196/84754 (PMC13153395; doi:10.2196/84754)
Supplement: Multimedia Appendix 1 [file mental-v13-e84754-s001.docx]

**Section A: Behaviour Change Techniques and Their BCTTv1 Definitions**

| **BCT** | **BCTTv1 Definition** |
| --- | --- |
| 1.1 Goal setting (behaviour) | Set or agree on a goal defined in terms of  the behaviour to be achieved |
| 1.2 Problem solving | Analyse, or prompt the person to analyse,  factors influencing the behaviour and  generate or select strategies that include  overcoming barriers and/or increasing  facilitators (includes ‘Relapse Prevention’  and ‘Coping Planning’) |
| 2.2 Feedback on behaviour | Monitor and provide informative or  evaluative feedback on performance of  the behaviour (e.g. form, frequency,  duration, intensity) |
| 2.3 Self-monitoring (behaviour) | Establish a method for the person to  monitor and record their behaviour(s) as  part of a behaviour change strategy |
| 3.1 Social support (unspecified) | Advise on, arrange or provide social  support (e.g. from friends, relatives,  colleagues,’ buddies’ or staff) or non-  contingent praise or reward for  performance of the behaviour. It includes  encouragement and counselling, but only  when it is directed at the behaviour |
| 3.2 Social support (practical) | Advise on, arrange, or provide practical  help (e.g. from friends, relatives,  colleagues, ‘buddies’ or staff) for  performance of the behaviour |
| 4.1 Instruction on how to perform a behaviour | Advise or agree on how to perform the  behaviour (includes ‘Skills training’) |
| 5.1 Information about health consequences | Provide information (e.g. written, verbal,  visual) about health consequences of  performing the behaviour |
| 6.1 Demonstration of the behaviour | Provide an observable sample of the  performance of the behaviour, directly in  person or indirectly e.g. via film, pictures,  for the person to aspire to or imitate |
| 6.2 Social comparison | Draw attention to others’ performance to  allow comparison with the person’s own  performance |
| 7.1 Prompts/cues | Introduce or define environmental or  social stimulus with the purpose of  prompting or cueing the behaviour. The  prompt or cue would normally occur at  the time or place of performance |
| 8.7 Graded Tasks | Set easy-to-perform tasks, making them  increasingly difficult, but achievable, until  behaviour is performed |
| 9.1 Credible Sources | Present verbal or visual communication  from a credible source in favour of or  against the behaviour |
| 9.2 Pros and Cons | Advise the person to identify and compare  reasons for wanting (pros) and not  wanting to (cons) change the behaviour  (includes ‘Decisional balance’) |
| 10.1 Material incentive (behaviour) | Inform that money, vouchers or other  valued objects will be delivered if and only  if there has been effort and/or progress in  performing the behaviour (includes  ‘Positive reinforcement’) |
| 10.3 Non-specific reward | Arrange delivery of a reward if and only if  there has been effort and/or progress in  performing the behaviour (includes  ‘Positive reinforcement’) |
| 10.10 Reward (outcome) | Arrange for the delivery of a reward if and  only if there has been effort and/or  progress in achieving the behavioural  outcome (includes ‘Positive  reinforcement’) |
| 10.11 Future Punishment | Inform that future punishment or removal  of reward will be a consequence of  performance of an unwanted behaviour  (may include fear arousal) (includes  ‘Threat’) |
| 12.1 Restructuring the physical environment | Change, or advise to change the physical  environment in order to facilitate  performance of the wanted behaviour or  create barriers to the unwanted behaviour  (other than prompts/cues, rewards and  punishments) |
| 12.5 Adding objects to the environment | Add objects to the environment in order  to facilitate performance of the behaviour |
| 15.3 Focus on past successes | Advise to think about or list previous  successes in performing the behaviour (or  parts of it) |

The Behaviour Change Technique labels, numbering, and definitions are sourced directly from the Behaviour Change Technique Taxonomy v1 (Michie et al. 2013).

**Section B: Search Strategy Used for PubMed**

PubMed

“Internet”[Mesh] OR “Mobile applications”[Mesh] OR “Computers”[Mesh] OR “Therapy, Computer-Assisted”[Mesh] OR “Telemedicine”[Mesh] OR “Mobile Applications”[Mesh] OR “Internet-Based Intervention”[Mesh] OR “Internet”[All Fields] OR “Mobile applications”[All Fields] OR “Computer”[All Fields] OR “Computer-assisted”[All Fields] OR “Telemedicine”[All Fields] OR “Mobile Applications”[All Fields] OR “Internet-Based Intervention”[All Fields] OR “Online”[All Fields] OR “Web”[All Fields] OR “E-health”[All Fields] OR “Mobile phone”[All Fields] OR “Smartphone”[All Fields] OR “Mobile device”[All Fields] OR “Tablet”[All Fields] OR “App”[All Fields] OR “CD-ROM”[All Fields] OR “digital health”[All Fields] OR “eHealth”[All Fields] OR “digital intervention”[All Fields] OR “online program”[All Fields] OR “web-based”[All Fields] OR “app-based”[All Fields] OR “mobile health”[All Fields] OR “SMS”[All Fields] OR “text messag*”[All Fields] OR “messenger”[All Fields]

"behaviour change"[All Fields] OR "behaviour change"[All Fields] OR "behavioural change"[All Fields] OR "behavioral change"[All Fields] OR "active ingredient*"[All Fields] OR "active element*"[All Fields] OR "intervention component*"[All Fields] OR "mechanism*"[All Fields] OR “Abraham”[All Fields] OR “Michie”[All Fields] OR “intervention function”[All Fields] OR “process variable”[All Fields] OR “therapeutic change”[All Fields] OR “indirect effect”[All Fields] OR “working function”[All Fields]

"smoking"[All Fields] OR "nicotine"[All Fields] OR "tobacco use"[All Fields] OR "smoking cessation"[All Fields] OR "cigarette smoking"[All Fields] OR "vaping"[All Fields] OR "e-cigarettes"[All Fields] OR "tobacco cessation"[All Fields] OR "nicotine dependence"[All Fields] OR "Smoking"[MeSH] OR "Tobacco Use Disorder"[MeSH] OR "Smoking Cessation"[MeSH] OR "Nicotine"[MeSH] OR "obesity"[All Fields] OR "overweight"[All Fields] OR "weight management"[All Fields] OR "body weight"[All Fields] OR "body mass index"[All Fields] OR "BMI"[All Fields] OR "obese"[All Fields] OR "weight reduction"[All Fields] OR "weight control"[All Fields] OR "Obesity"[MeSH] OR "Body Weight"[MeSH] OR "Overweight"[MeSH] OR "Weight Loss"[MeSH] OR "diet"[All Fields] OR "dietary habits"[All Fields] OR "nutrition"[All Fields] OR "nutritional intervention"[All Fields] OR "eating habits"[All Fields] OR "food"[All Fields] OR "diet therapy"[All Fields] OR "dietary patterns"[All Fields] OR "healthy diet"[All Fields] OR "Diet"[MeSH] OR "Diet Therapy"[MeSH] OR "Feeding Behavior"[MeSH] OR "Nutritional Status"[MeSH] OR "alcohol"[All Fields] OR "drinking"[All Fields] OR "alcohol consumption"[All Fields] OR "alcoholism"[All Fields] OR "alcoholic beverages"[All Fields] OR "binge drinking"[All Fields] OR "alcohol abuse"[All Fields] OR "alcohol use disorder" OR "Alcohol Drinking"[MeSH] OR "Alcoholism"[MeSH] OR "Alcoholic Beverages"[MeSH] OR "Binge Drinking"[MeSH] OR "physical activity"[All Fields] OR "exercise"[All Fields] OR "fitness program"[All Fields] OR "physical fitness"[All Fields] OR "aerobic exercise"[All Fields] OR "sports"[All Fields] OR "physical exertion"[All Fields] OR "sedentary lifestyle"[All Fields] OR "active lifestyle"[All Fields] AND "Exercise"[MeSH] OR "Physical Fitness"[MeSH] OR "Physical Exertion"[MeSH] OR "Sedentary Behavior"[MeSH]

**Section C: AMSTAR-2 Quality Assessment Summary**


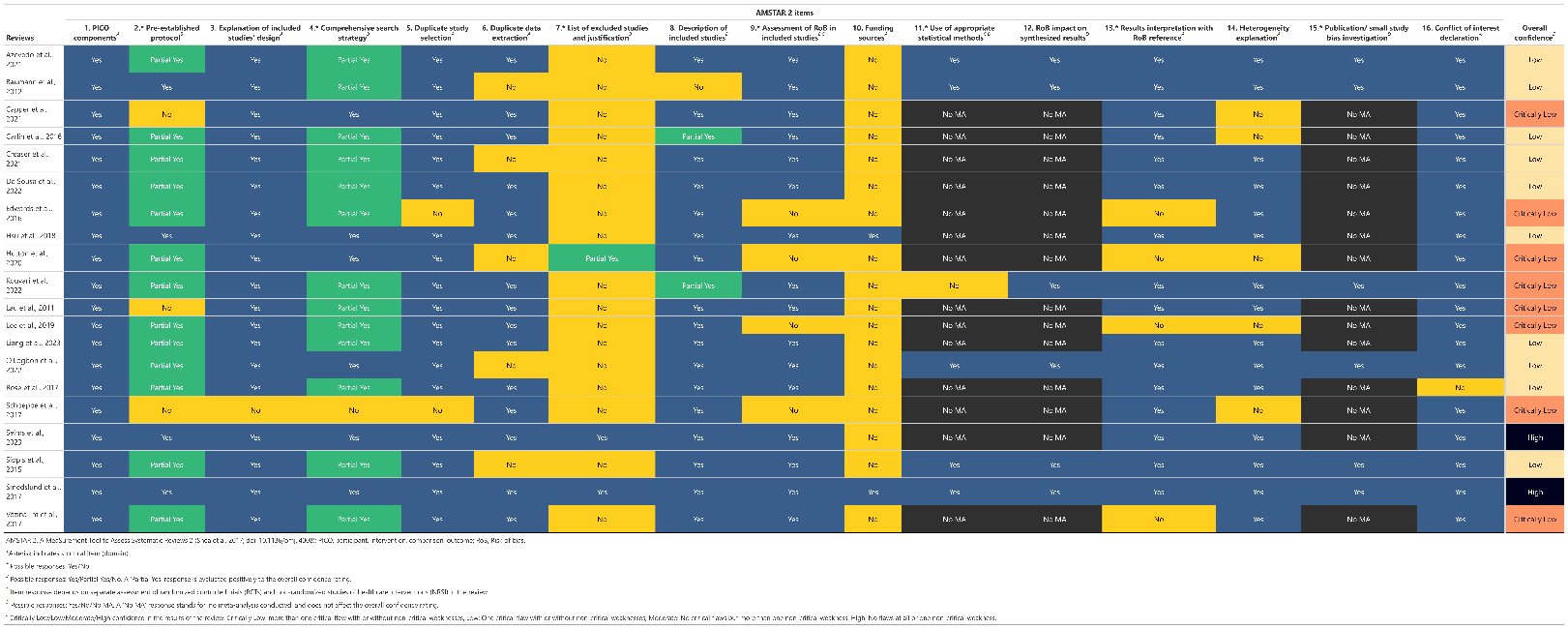


**Section D: Primary Study Overlap Across Included Systematic Reviews**

This appendix provides a systematic quantification of primary study overlap across all 20 systematic reviews included in the umbrella review. Overlap in umbrella reviews occurs when the same primary study is included by more than one eligible systematic review, which can lead to over-representation of specific findings and potentially inflate estimates of BCT effectiveness.

Two of the 20 included reviews, Schoeppe et al. (2017) and Edwards et al. (2016), assessed the content of commercial smartphone applications and did not include primary intervention studies with participant-level data. These reviews are therefore excluded from the overlap analysis but retained in Table D1 for completeness. The remaining 18 reviews contributed a combined total of 302 unique primary studies.

**Table D1.** Included systematic reviews and review IDs used in the overlap analysis

| **ID** | **Reference** |
| --- | --- |
| **R1** | Azevedo et al. (2022) [32] |
| **R2** | Baumann et al. (2022) [29] |
| **R3** | Capper et al. (2022) [37] |
| **R4** | Vézina-Im et al. (2017) [42] |
| **R5** | Carlin et al. (2016) [28] |
| **R6** | Creaser et al. (2021) [31] |
| **R7** | de Sousa et al. (2022) [2] |
| **R8** | Hsu et al. (2018) [15] |
| **R9** | Hutton et al. (2020) [39] |
| **R10** | Kouvari et al. (2022) [40] |
| **R11** | Lau et al. (2011) [35] |
| **R12** | Lee et al. (2019) [36] |
| **R13** | Liang et al. (2023) [30] |
| **R14** | Rose et al. (2017) [6] |
| **R15** | O'Logbon et al. (2024) [33] |
| **R16** | Smedslund et al. (2017) [27] |
| **R17** | Seims et al. (2023) [26] |
| **R18** | Siopis et al. (2015) [34] |
| **R19** | Schoeppe et al. (2017) [41] |
| **R20** | Edwards et al. (2016) [38] |

**Table D2.** Primary studies included in two or more reviews across the included systematic reviews (N=37)

The table below lists all 37 primary studies that appear in more than one included systematic review, along with the reviews in which they are included. Studies are sorted by the number of reviews in which they appear (descending). Cells in bold indicate studies appearing in three or more reviews.

| **Primary study** | **Reviews including this study** | **Reviews, n** |
| --- | --- | --- |
| Whittemore et al. (2013) | R4 – Vézina-Im et al. (2017) [42], R8 – Hsu et al. (2018) [15], R12 – Lee et al. (2019) [36], R14 – Rose et al. (2017) [6] | **4** |
| Chen et al. (2019) | R1 – Azevedo et al. (2022) [32], R2 – Baumann et al. (2022) [29], R10 – Kouvari et al. (2022) [40] | **3** |
| Cullen et al. (2013) | R8 – Hsu et al. (2018) [15], R12 – Lee et al. (2019) [36], R14 – Rose et al. (2017) [6] | **3** |
| de Niet et al. (2012) | R1 – Azevedo et al. (2022) [32], R10 – Kouvari et al. (2022) [40], R18 – Siopis et al. (2015) [34] | **3** |
| Direito et al. (2015) | R2 – Baumann et al. (2022) [29], R12 – Lee et al. (2019) [36], R14 – Rose et al. (2017) [6]R14 – Rose et al. (2017) | **3** |
| Gaudet et al. (2017) | R2 – Baumann et al. (2022) [29], R6 – Creaser et al. (2021) [31], R12 – Lee et al. (2019) [36] | **3** |
| Guthrie et al. (2015) | R6 – Creaser et al. (2021) [31], R12 – Lee et al. (2019) [36], R14 – Rose et al. (2017) [6] | **3** |
| Jones et al. (2014) | R4 – Vézina-Im et al. (2017) [42], R8 – Hsu et al. (2018) [15], R14 – Rose et al. (2017) [6] | **3** |
| Mendoza et al. (2017) | R2 – Baumann et al. (2022) [29], R6 – Creaser et al. (2021) [31], R12 – Lee et al. (2019) [36] | **3** |
| Patrick et al. (2013) | R1 – Azevedo et al. (2022) [32], R12 – Lee et al. (2019) [36], R14 – Rose et al. (2017) [6] | **3** |
| Slootmaker et al. (2010) | R6 – Creaser et al. (2021) [31], R12 – Lee et al. (2019) [36], R14 – Rose et al. (2017) [6] | **3** |
| Smith et al. (2014) | R4 – Vézina-Im et al. (2017) [42], R12 – Lee et al. (2019) [36], R14 – Rose et al. (2017) [6] | **3** |
| Sousa et al. (2015) | R8 – Hsu et al. (2018) [15], R12 – Lee et al. (2019) [36], R14 – Rose et al. (2017) [6] | **3** |
| Staiano et al. (2018) | R1 – Azevedo et al. (2022) [32], R10 – Kouvari et al. (2022) [40], R17 – Seims et al. (2023) [26] | **3** |
| Williamson et al. (2006) | R1 – Azevedo et al. (2022) [32], R10 – Kouvari et al. (2022) [40], R11 – Lau et al. (2011) [35] | **3** |
| Bertholet et al. (2015) | R15 – O'Logbon et al. (2024) [33], R16 – Smedslund et al. (2017) [27] | 2 |
| Doumas et al. (2009) | R15 – O'Logbon et al. (2024) [33], R16 – Smedslund et al. (2017) [27] | 2 |
| Gajecki et al. (2014) | R9 – Hutton et al. (2020) [39], R16 – Smedslund et al. (2017) [27] | 2 |
| Jago et al. (2006) | R11 – Lau et al. (2011) [35], R14 – Rose et al. (2017) [6] | 2 |
| Kypri et al. (2004) | R15 – O'Logbon et al. (2024) [33], R16 – Smedslund et al. (2017) [27] | 2 |
| Kypri et al. (2009) | R15 – O'Logbon et al. (2024) [33], R16 – Smedslund et al. (2017) [27] | 2 |
| Lana et al. (2014) | R8 – Hsu et al. (2018) [15], R14 – Rose et al. (2017) [6] | 2 |
| Larsen et al. (2018) | R7 – de Sousa et al. (2022) [2], R12 – Lee et al. (2019) [36] | 2 |
| Lau et al. (2012) | R12 – Lee et al. (2019) [36], R14 – Rose et al. (2017) [6] | 2 |
| Lubans et al. (2009) | R11 – Lau et al. (2011) [35], R14 – Rose et al. (2017) [6] | 2 |
| Maddison et al. (2011) | R1 – Azevedo et al. (2022) [32], R17 – Seims et al. (2023) [26] | 2 |
| Mason et al. (2014) | R9 – Hutton et al. (2020) [39], R16 – Smedslund et al. (2017) [27] | 2 |
| Newton et al. (2009) | R11 – Lau et al. (2011) [35], R14 – Rose et al. (2017) [6] | 2 |
| Nguyen et al. (2013) | R10 – Kouvari et al. (2022) [40], R18 – Siopis et al. (2015) [34] | 2 |
| Schoenfelder et al. (2017) | R6 – Creaser et al. (2021) [31], R12 – Lee et al. (2019) [36] | 2 |
| Sirriyeh et al. (2010) | R2 – Baumann et al. (2022) [29], R14 – Rose et al. (2017) [6] | 2 |
| Suffoletto et al. (2012) | R9 – Hutton et al. (2020) [39], R16 – Smedslund et al. (2017) [27] | 2 |
| Suffoletto et al. (2015) | R15 – O'Logbon et al. (2024) [33], R16 – Smedslund et al. (2017) [27] | 2 |
| Walters et al. (2007) | R15 – O'Logbon et al. (2024) [33], R16 – Smedslund et al. (2017) [27] | 2 |
| Weitzel et al. (2007) | R9 – Hutton et al. (2020) [39], R16 – Smedslund et al. (2017) [27] | 2 |
| Williamson et al. (2005) | R10 – Kouvari et al. (2022) [40], R14 – Rose et al. (2017) [6] | 2 |
| Witkiewitz et al. (2014) | R15 – O'Logbon et al. (2024) [33], R16 – Smedslund et al. (2017) [27] | 2 |
