# Supplementary material for: Behavior Change Techniques in Digital Health Interventions for Promoting Adolescent Health Behaviors: Systematic Umbrella Review
Source: JMIR Ment Health. 2026 May 6;13:e84754. doi: 10.2196/84754 (PMC13153395; doi:10.2196/84754)
Supplement: Multimedia Appendix 2 [file mental-v13-e84754-s002.docx]

**Table S1.** Summary of the effectiveness of health-related behaviour change techniques across health domains.

| BCTs  Reviews (n=20) | 1.1 Goal setting (behaviour) | 1.2 Problem solving | 2.2 Feedback on behaviour | 2.3 Self-monitoring (behaviour) | 3.1 Social support (unspecified) | 3.2 Social support (practical) | 4.1 Instruction on how to perform a behaviour | 5.1 Information about health consequences | 6.1 Demonstration of the behaviour | 6.2 Social comparison | 7.1 Prompts/cues | 8.7 Graded Tasks | 9.1 Credible Sources | 9.2 Pros and Cons | 10.1 Material incentive (behaviour) | 10.3 Non-specific reward | 10.10 Reward (outcome) | 10.11 Future Punishment | 12.1 Restructuring the physical environment | 12.5 Adding objects to the environment | 15.3 Focus on past successes |
| --- | --- | --- | --- | --- | --- | --- | --- | --- | --- | --- | --- | --- | --- | --- | --- | --- | --- | --- | --- | --- | --- |
| Physical Activity |  |  |  |  |  |  |  |  |  |  |  |  |  |  |  |  |  |  |  |  |  |
| Carlin et al. (2016) [28] | Ø |  | Ø | Ø | O+ |  |  |  |  |  |  |  |  |  |  |  |  |  |  | O+ |  |
| Baumann et al. 2022 [29] |  |  | ☑ | ☑ | ☑ |  |  |  |  | ☑ | ☑ |  |  |  |  |  | ☑ | ☑ |  |  |  |
| Seims et al. 2023 [26] | ☑ |  | ☑ | ☑ | O+ | ☑ | ☑ |  |  |  |  | ☑ |  |  |  |  |  |  |  | ☑ |  |
| Lau et al. (2011) [35] | ☑ | ☑ | ☑ | ☑ | ☑ |  | ☑ | ☑ | ☑ |  |  |  |  |  |  |  | ☑ | ☑ |  |  |  |
| Liang et al. (2023) [30] | ☑ |  |  | ☑ | ☑ |  |  |  |  |  | ☑ |  |  |  |  | ☑ |  |  | ☑ | ☑ |  |
| Creaser et al. (2021) [31] | Ø |  | Ø | Ø | Ø |  | Ø |  |  | Ø | Ø |  |  |  |  |  | Ø |  |  | Ø |  |
| Lee et al. (2019) [36] | ☑ |  | ☑ | ☑ | ☑ | ☑ |  | ☑ | ☑ |  | ☑ |  |  |  |  | ☑ |  |  |  | ☑ |  |
| Dietary Habits |  |  |  |  |  |  |  |  |  |  |  |  |  |  |  |  |  |  |  |  |  |
| Hsu et al. (2018) [15] | ☑ |  | ☑ | ☑ | ☑ |  | ☑ |  | ☑ |  |  |  |  |  | ☑ | ☑ |  |  |  |  |  |
| Capper et al. (2022) [37] | ☑ |  | O+ | O+ | O+ |  | ☑ | ☑ |  |  |  |  |  | ☑ |  |  |  | ☑ | O+ |  |  |
| Vézina-Im et al. (2017) [42] | ☑ |  |  | ☑ | ☑ |  |  | ☑ |  |  |  |  |  | ☑ |  |  |  | ☑ | ☑ |  |  |
| Alcohol Consumption |  |  |  |  |  |  |  |  |  |  |  |  |  |  |  |  |  |  |  |  |  |
| Hutton et al. (2020) [39] |  |  | ☑ |  | ☑ |  |  |  | ☑ |  |  |  |  |  |  |  |  |  |  |  |  |
| Smedslund et al. (2017) [27] | ☑ | ☑ | ▲ |  | ☑ |  | ☑ | ☑ |  |  |  |  | ☑ | ☑ |  |  |  |  |  |  |  |
| O'Logbon et al. (2024) [33] | ☑ |  | Ø | Ø |  |  |  |  | ☑ |  |  |  |  |  |  |  |  |  |  |  |  |
| Obesity Management |  |  |  |  |  |  |  |  |  |  |  |  |  |  |  |  |  |  |  |  |  |
| Azevedo et al. (2022) [32] | O+ | O+ | ☑ | O+ | O+ |  | ☑ | ☑ | ☑ |  | ☑ |  |  |  | ☑ | O+ |  |  |  | O+ |  |
| Nicotine use |  |  |  |  |  |  |  |  |  |  |  |  |  |  |  |  |  |  |  |  |  |
| O'Logbon et al. (2024) [33] |  |  |  |  | O+ |  |  | O+ |  |  |  |  |  |  |  |  |  |  |  |  |  |
| Physical Activity, Dietary Habits, Obesity Management |  |  |  |  |  |  |  |  |  |  |  |  |  |  |  |  |  |  |  |  |  |
| Siopis et al. (2015) [34] | ☑ |  |  | ☑ | ☑ |  |  |  |  |  |  |  |  |  |  |  |  |  |  | ☑ |  |
| Kouvari et al. (2022) [40] | ☑ |  | ☑ | O+ | ▲ | ▲ | ☑ | ☑ |  |  | ☑ |  | ☑ |  |  | ☑ |  |  |  | ☑ |  |
| Physical Activity and Dietary Habits |  |  |  |  |  |  |  |  |  |  |  |  |  |  |  |  |  |  |  |  |  |
| Rose et al. (2017) [6] | O+ |  |  | O+ | O+ |  | O+ | O+ |  |  | ☑ | ☑ |  |  |  | ☑ |  |  |  |  |  |
| Schoeppe et al. (2017) [41] |  |  | ☑ | ☑ | ☑ |  | ☑ | ☑ |  | ☑ | ☑ |  |  |  |  | ☑ | ☑ |  | ☑ |  |  |
| Alcohol Consumption, Nicotine Use, Physical Activity, Dietary Habits |  |  |  |  |  |  |  |  |  |  |  |  |  |  |  |  |  |  |  |  |  |
| De Sousa et al. (2022) [2] | ☑ |  | ☑ | ☑ | ☑ |  |  | ☑ |  |  | ☑ | ☑ |  |  |  |  |  |  |  | ☑ |  |
| Nicotine Use, Physical Activity, Dietary Habits |  |  |  |  |  |  |  |  |  |  |  |  |  |  |  |  |  |  |  |  |  |
| Edwards et al. (2016) [38] | ☑ | ☑ | ☑ | ☑ | ☑ |  |  |  |  |  |  |  |  |  | ☑ | ☑ | ☑ | ☑ | ☑ | ☑ | ☑ |

Classification of effectiveness from current systematic reviews and meta-analyses:

▲ Positive effects of the BCT reported by the study based on quantitative and statistical analyses (meta-regression, subgroup analyses).
O+ Positive effects of the BCT based on qualitative analyses, reported effectiveness of the included / extracted studies or only on frequency analyses.

▼ Negative effects based on quantitative and statistical analyses (meta-regression, subgroup analyses).

O− Negative effects of the BCT based on qualitative analyses, reported effectiveness of the included / extracted studies or only on frequency analyses.

Ø Mixed evidence or No effect.

☑ BCTs identified or reported where the authors did not conduct any analysis on effectiveness.
